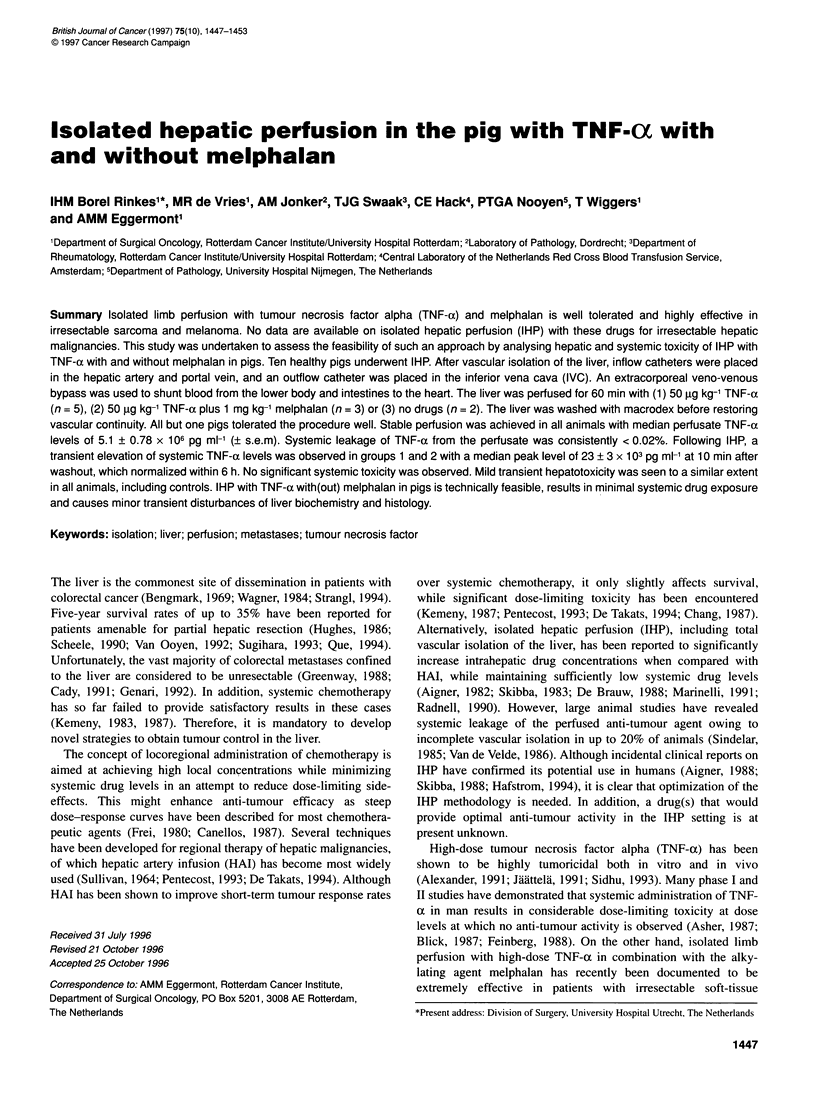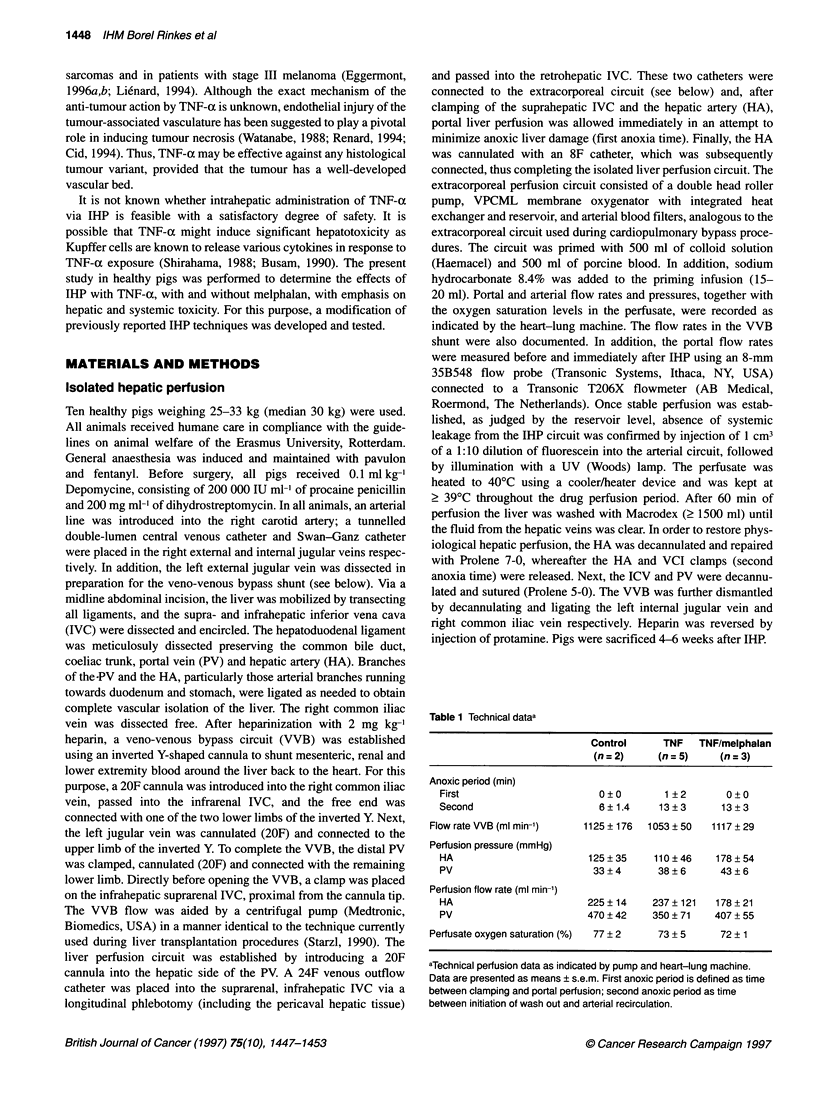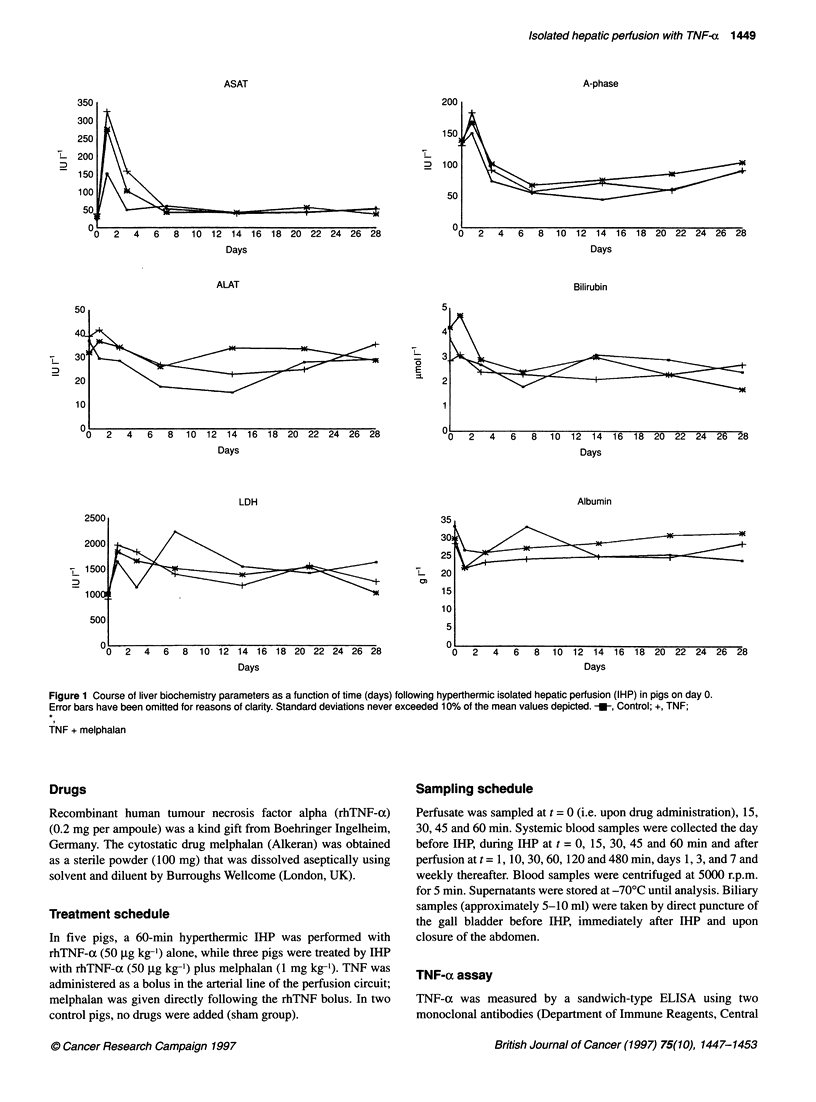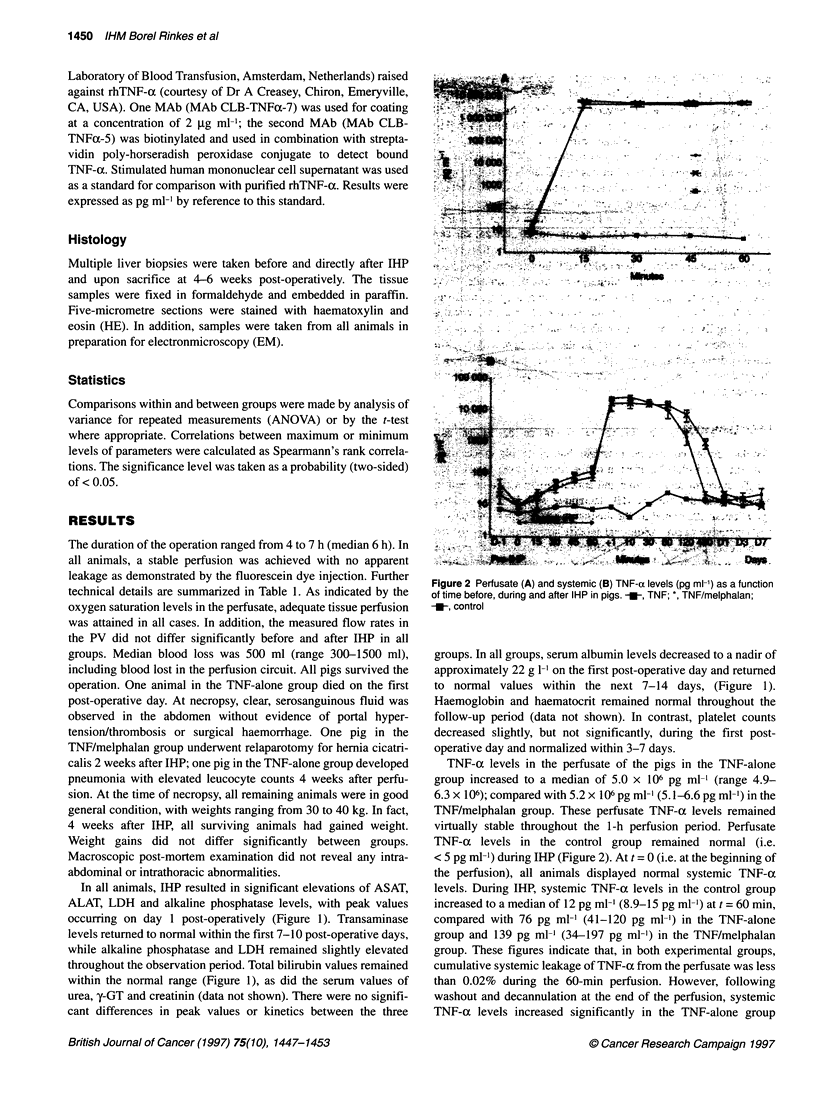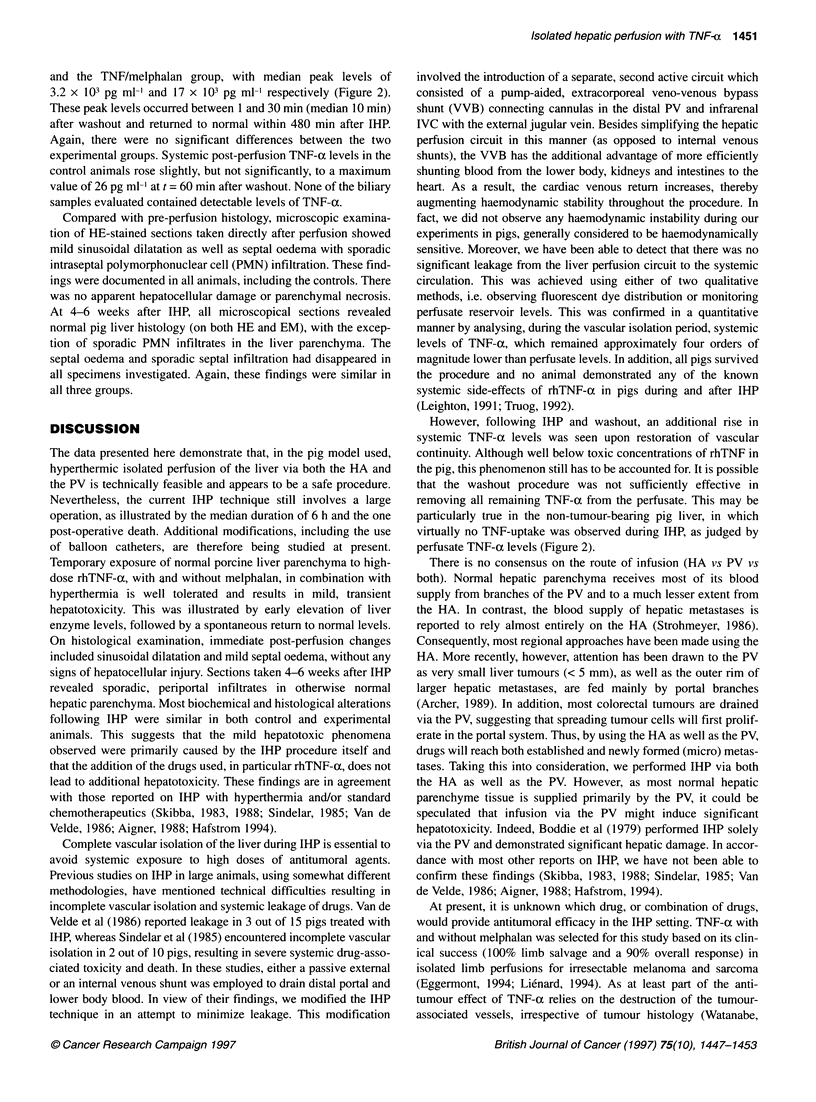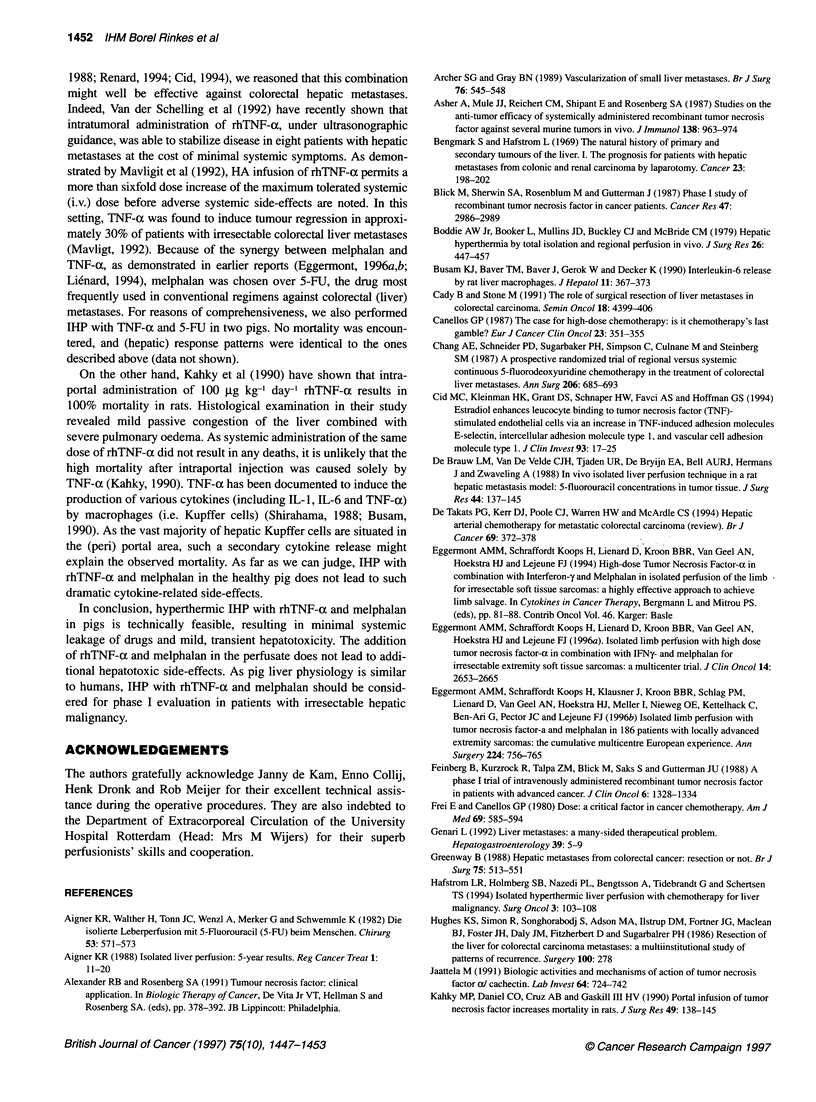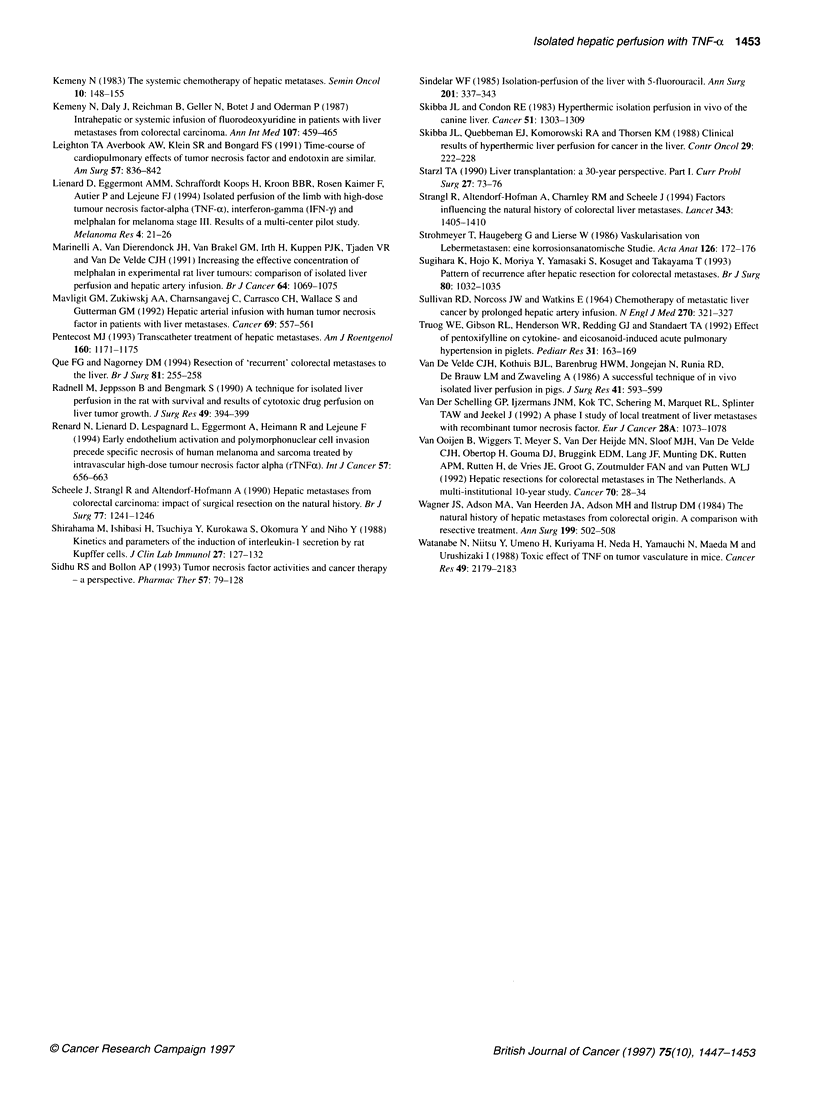# Isolated hepatic perfusion in the pig with TNF-alpha with and without melphalan.

**DOI:** 10.1038/bjc.1997.248

**Published:** 1997

**Authors:** I. H. Borel Rinkes, M. R. de Vries, A. M. Jonker, T. J. Swaak, C. E. Hack, P. T. Nooyen, T. Wiggers, A. M. Eggermont

**Affiliations:** Department of Surgical Oncology, Rotterdam Cancer Institute/University Hospital Rotterdam, The Netherlands.

## Abstract

Isolated limb perfusion with tumour necrosis factor alpha (TNF-alpha) and melphalan is well tolerated and highly effective in irresectable sarcoma and melanoma. No data are available on isolated hepatic perfusion (IHP) with these drugs for irresectable hepatic malignancies. This study was undertaken to assess the feasibility of such an approach by analysing hepatic and systemic toxicity of IHP with TNF-alpha with and without melphalan in pigs. Ten healthy pigs underwent IHP. After vascular isolation of the liver, inflow catheters were placed in the hepatic artery and portal vein, and an outflow catheter was placed in the inferior vena cava (IVC). An extracorporeal veno-venous bypass was used to shunt blood from the lower body and intestines to the heart. The liver was perfused for 60 min with (1) 50 microg kg(-1) TNF-alpha (n = 5), (2) 50 microg kg(-1) TNF-alpha plus 1 mg kg(-1) melphalan (n = 3) or (3) no drugs (n = 2). The liver was washed with macrodex before restoring vascular continuity. All but one pigs tolerated the procedure well. Stable perfusion was achieved in all animals with median perfusate TNF-alpha levels of 5.1 +/- 0.78 x 10(6) pg ml(-1) (+/- s.e.m). Systemic leakage of TNF-alpha from the perfusate was consistently < 0.02%. Following IHP, a transient elevation of systemic TNF-alpha levels was observed in groups 1 and 2 with a median peak level of 23 +/- 3 x 10(3) pg ml(-1) at 10 min after washout, which normalized within 6 h. No significant systemic toxicity was observed. Mild transient hepatotoxicity was seen to a similar extent in all animals, including controls. IHP with TNF-alpha with(out) melphalan in pigs is technically feasible, results in minimal systemic drug exposure and causes minor transient disturbances of liver biochemistry and histology.


British Journal of Cancer (1997) 75(10), 1447–1453
© 1997 Cancer Research Campaign

### Isolated hepatic perfusion in the pig with TNF-α with and without melphalan

**IHM Borel Rinkes[1]*, MR de Vries[1], AM Jonker[2], TJG Swaak[3], CE Hack[4], PTGA Nooyen[5], T Wiggers[1] and AMM Eggermont[1]**

[1]Department of Surgical Oncology, Rotterdam Cancer Institute/University Hospital Rotterdam; [2]Laboratory of Pathology, Dordrecht; [3]Department of Rheumatology, Rotterdam Cancer Institute/University Hospital Rotterdam; [4]Central Laboratory of the Netherlands Red Cross Blood Transfusion Service, Amsterdam; [5]Department of Pathology, University Hospital Nijmegen, The Netherlands

**Summary** Isolated limb perfusion with tumour necrosis factor alpha (TNF-α) and melphalan is well tolerated and highly effective in irresectable sarcoma and melanoma. No data are available on isolated hepatic perfusion (IHP) with these drugs for irresectable hepatic malignancies. This study was undertaken to assess the feasibility of such an approach by analysing hepatic and systemic toxicity of IHP with TNF-α with and without melphalan in pigs. Ten healthy pigs underwent IHP. After vascular isolation of the liver, inflow catheters were placed in the hepatic artery and portal vein, and an outflow catheter was placed in the inferior vena cava (IVC). An extracorporeal veno-venous bypass was used to shunt blood from the lower body and intestines to the heart. The liver was perfused for 60 min with (1) 50 μg kg$^{-1}$ TNF-α ($n$ = 5), (2) 50 μg kg$^{-1}$ TNF-α plus 1 mg kg$^{-1}$ melphalan ($n$ = 3) or (3) no drugs ($n$ = 2). The liver was washed with macrodex before restoring vascular continuity. All but one pigs tolerated the procedure well. Stable perfusion was achieved in all animals with median perfusate TNF-α levels of 5.1 ± 0.78 × 10$^6$ pg ml$^{-1}$ (± s.e.m). Systemic leakage of TNF-α from the perfusate was consistently < 0.02%. Following IHP, a transient elevation of systemic TNF-α levels was observed in groups 1 and 2 with a median peak level of 23 ± 3 × 10$^3$ pg ml$^{-1}$ at 10 min after washout, which normalized within 6 h. No significant systemic toxicity was observed. Mild transient hepatotoxicity was seen to a similar extent in all animals, including controls. IHP with TNF-α with(out) melphalan in pigs is technically feasible, results in minimal systemic drug exposure and causes minor transient disturbances of liver biochemistry and histology.

**Keywords:** isolation; liver; perfusion; metastases; tumour necrosis factor

The liver is the commonest site of dissemination in patients with colorectal cancer (Bengmark, 1969; Wagner, 1984; Strangl, 1994). Five-year survival rates of up to 35% have been reported for patients amenable for partial hepatic resection (Hughes, 1986; Scheele, 1990; Van Ooyen, 1992; Sugihara, 1993; Que, 1994). Unfortunately, the vast majority of colorectal metastases confined to the liver are considered to be unresectable (Greenway, 1988; Cady, 1991; Genari, 1992). In addition, systemic chemotherapy has so far failed to provide satisfactory results in these cases (Kemeny, 1983, 1987). Therefore, it is mandatory to develop novel strategies to obtain tumour control in the liver.

The concept of locoregional administration of chemotherapy is aimed at achieving high local concentrations while minimizing systemic drug levels in an attempt to reduce dose-limiting side-effects. This might enhance anti-tumour efficacy as steep dose–response curves have been described for most chemotherapeutic agents (Frei, 1980; Canellos, 1987). Several techniques have been developed for regional therapy of hepatic malignancies, of which hepatic artery infusion (HAI) has become most widely used (Sullivan, 1964; Pentecost, 1993; De Takats, 1994). Although HAI has been shown to improve short-term tumour response rates over systemic chemotherapy, it only slightly affects survival, while significant dose-limiting toxicity has been encountered (Kemeny, 1987; Pentecost, 1993; De Takats, 1994; Chang, 1987). Alternatively, isolated hepatic perfusion (IHP), including total vascular isolation of the liver, has been reported to significantly increase intrahepatic drug concentrations when compared with HAI, while maintaining sufficiently low systemic drug levels (Aigner, 1982; Skibba, 1983; De Brauw, 1988; Marinelli, 1991; Radnell, 1990). However, large animal studies have revealed systemic leakage of the perfused anti-tumour agent owing to incomplete vascular isolation in up to 20% of animals (Sindelar, 1985; Van de Velde, 1986). Although incidental clinical reports on IHP have confirmed its potential use in humans (Aigner, 1988; Skibba, 1988; Hafstrom, 1994), it is clear that optimization of the IHP methodology is needed. In addition, a drug(s) that would provide optimal anti-tumour activity in the IHP setting is at present unknown.

High-dose tumour necrosis factor alpha (TNF-α) has been shown to be highly tumoricidal both in vitro and in vivo (Alexander, 1991; Jäättelä, 1991; Sidhu, 1993). Many phase I and II studies have demonstrated that systemic administration of TNF-α in man results in considerable dose-limiting toxicity at dose levels at which no anti-tumour activity is observed (Asher, 1987; Blick, 1987; Feinberg, 1988). On the other hand, isolated limb perfusion with high-dose TNF-α in combination with the alkylating agent melphalan has recently been documented to be extremely effective in patients with irresectable soft-tissue

*Received 31 July 1996*
*Revised 21 October 1996*
*Accepted 25 October 1996*

*Correspondence to:* AMM Eggermont, Rotterdam Cancer Institute, Department of Surgical Oncology, PO Box 5201, 3008 AE Rotterdam, The Netherlands

*Present address: Division of Surgery, University Hospital Utrecht, The Netherlands sarcomas and in patients with stage III melanoma (Eggermont, 1996*a,b*; Liénard, 1994). Although the exact mechanism of the anti-tumour action by TNF-α is unknown, endothelial injury of the tumour-associated vasculature has been suggested to play a pivotal role in inducing tumour necrosis (Watanabe, 1988; Renard, 1994; Cid, 1994). Thus, TNF-α may be effective against any histological tumour variant, provided that the tumour has a well-developed vascular bed.

It is not known whether intrahepatic administration of TNF-α via IHP is feasible with a satisfactory degree of safety. It is possible that TNF-α might induce significant hepatotoxicity as Kupffer cells are known to release various cytokines in response to TNF-α exposure (Shirahama, 1988; Busam, 1990). The present study in healthy pigs was performed to determine the effects of IHP with TNF-α, with and without melphalan, with emphasis on hepatic and systemic toxicity. For this purpose, a modification of previously reported IHP techniques was developed and tested.

### MATERIALS AND METHODS

### Isolated hepatic perfusion

Ten healthy pigs weighing 25–33 kg (median 30 kg) were used. All animals received humane care in compliance with the guidelines on animal welfare of the Erasmus University, Rotterdam. General anaesthesia was induced and maintained with pavulon and fentanyl. Before surgery, all pigs received 0.1 ml $kg^{-1}$ Depomycine, consisting of 200 000 IU $ml^{-1}$ of procaine penicillin and 200 mg $ml^{-1}$ of dihydrostreptomycin. In all animals, an arterial line was introduced into the right carotid artery; a tunnelled double-lumen central venous catheter and Swan–Ganz catheter were placed in the right external and internal jugular veins respectively. In addition, the left external jugular vein was dissected in preparation for the veno-venous bypass shunt (see below). Via a midline abdominal incision, the liver was mobilized by transecting all ligaments, and the supra- and infrahepatic inferior vena cava (IVC) were dissected and encircled. The hepatoduodenal ligament was meticulosuly dissected preserving the common bile duct, coeliac trunk, portal vein (PV) and hepatic artery (HA). Branches of the PV and the HA, particularly those arterial branches running towards duodenum and stomach, were ligated as needed to obtain complete vascular isolation of the liver. The right common iliac vein was dissected free. After heparinization with 2 mg $kg^{-1}$ heparin, a veno-venous bypass circuit (VVB) was established using an inverted Y-shaped cannula to shunt mesenteric, renal and lower extremity blood around the liver back to the heart. For this purpose, a 20F cannula was introduced into the right common iliac vein, passed into the infrarenal IVC, and the free end was connected with one of the two lower limbs of the inverted Y. Next, the left jugular vein was cannulated (20F) and connected to the upper limb of the inverted Y. To complete the VVB, the distal PV was clamped, cannulated (20F) and connected with the remaining lower limb. Directly before opening the VVB, a clamp was placed on the infrahepatic suprarenal IVC, proximal from the cannula tip. The VVB flow was aided by a centrifugal pump (Medtronic, Biomedics, USA) in a manner identical to the technique currently used during liver transplantation procedures (Starzl, 1990). The liver perfusion circuit was established by introducing a 20F cannula into the hepatic side of the PV. A 24F venous outflow catheter was placed into the suprarenal, infrahepatic IVC via a longitudinal phlebotomy (including the pericaval hepatic tissue) and passed into the retrohepatic IVC. These two catheters were connected to the extracorporeal circuit (see below) and, after clamping of the suprahepatic IVC and the hepatic artery (HA), portal liver perfusion was allowed immediately in an attempt to minimize anoxic liver damage (first anoxia time). Finally, the HA was cannulated with an 8F catheter, which was subsequently connected, thus completing the isolated liver perfusion circuit. The extracorporeal perfusion circuit consisted of a double head roller pump, VPCML membrane oxygenator with integrated heat exchanger and reservoir, and arterial blood filters, analogous to the extracorporeal circuit used during cardiopulmonary bypass procedures. The circuit was primed with 500 ml of colloid solution (Haemacel) and 500 ml of porcine blood. In addition, sodium hydrocarbonate 8.4% was added to the priming infusion (15–20 ml). Portal and arterial flow rates and pressures, together with the oxygen saturation levels in the perfusate, were recorded as indicated by the heart–lung machine. The flow rates in the VVB shunt were also documented. In addition, the portal flow rates were measured before and immediately after IHP using an 8-mm 35B548 flow probe (Transonic Systems, Ithaca, NY, USA) connected to a Transonic T206X flowmeter (AB Medical, Roermond, The Netherlands). Once stable perfusion was established, as judged by the reservoir level, absence of systemic leakage from the IHP circuit was confirmed by injection of 1 $cm^3$ of a 1:10 dilution of fluorescein into the arterial circuit, followed by illumination with a UV (Woods) lamp. The perfusate was heated to 40°C using a cooler/heater device and was kept at ≥ 39°C throughout the drug perfusion period. After 60 min of perfusion the liver was washed with Macrodex (≥ 1500 ml) until the fluid from the hepatic veins was clear. In order to restore physiological hepatic perfusion, the HA was decannulated and repaired with Prolene 7-0, whereafter the HA and VCI clamps (second anoxia time) were released. Next, the ICV and PV were decannulated and sutured (Prolene 5-0). The VVB was further dismantled by decannulating and ligating the left internal jugular vein and right common iliac vein respectively. Heparin was reversed by injection of protamine. Pigs were sacrificed 4–6 weeks after IHP.

**Table 1** Technical data[a]

| | Control (*n* = 2) | TNF (*n* = 5) | TNF/melphalan (*n* = 3) |
|---|---|---|---|
| Anoxic period (min) | | | |
| First | 0 ± 0 | 1 ± 2 | 0 ± 0 |
| Second | 6 ± 1.4 | 13 ± 3 | 13 ± 3 |
| Flow rate VVB (ml $min^{-1}$) | 1125 ± 176 | 1053 ± 50 | 1117 ± 29 |
| Perfusion pressure (mmHg) | | | |
| HA | 125 ± 35 | 110 ± 46 | 178 ± 54 |
| PV | 33 ± 4 | 38 ± 6 | 43 ± 6 |
| Perfusion flow rate (ml $min^{-1}$) | | | |
| HA | 225 ± 14 | 237 ± 121 | 178 ± 21 |
| PV | 470 ± 42 | 350 ± 71 | 407 ± 55 |
| Perfusate oxygen saturation (%) | 77 ± 2 | 73 ± 5 | 72 ± 1 |

[a]Technical perfusion data as indicated by pump and heart–lung machine. Data are presented as means ± s.e.m. First anoxic period is defined as time between clamping and portal perfusion; second anoxic period as time between initiation of wash out and arterial recirculation.

© Cancer Research Campaign 1997

**Figure 1** Course of liver biochemistry parameters as a function of time (days) following hyperthermic isolated hepatic perfusion (IHP) in pigs on day 0. Error bars have been omitted for reasons of clarity. Standard deviations never exceeded 10% of the mean values depicted. -■-, Control; +, TNF; *, TNF + melphalan

### Drugs

Recombinant human tumour necrosis factor alpha (rhTNF-α) (0.2 mg per ampoule) was a kind gift from Boehringer Ingelheim, Germany. The cytostatic drug melphalan (Alkeran) was obtained as a sterile powder (100 mg) that was dissolved aseptically using solvent and diluent by Burroughs Wellcome (London, UK).

### Treatment schedule

In five pigs, a 60-min hyperthermic IHP was performed with rhTNF-α (50 μg $kg^{-1}$) alone, while three pigs were treated by IHP with rhTNF-α (50 μg $kg^{-1}$) plus melphalan (1 mg $kg^{-1}$). TNF was administered as a bolus in the arterial line of the perfusion circuit; melphalan was given directly following the rhTNF bolus. In two control pigs, no drugs were added (sham group).

### Sampling schedule

Perfusate was sampled at $t = 0$ (i.e. upon drug administration), 15, 30, 45 and 60 min. Systemic blood samples were collected the day before IHP, during IHP at $t = 0$, 15, 30, 45 and 60 min and after perfusion at $t = 1$, 10, 30, 60, 120 and 480 min, days 1, 3, and 7 and weekly thereafter. Blood samples were centrifuged at 5000 r.p.m. for 5 min. Supernatants were stored at –70°C until analysis. Biliary samples (approximately 5–10 ml) were taken by direct puncture of the gall bladder before IHP, immediately after IHP and upon closure of the abdomen.

### TNF-α assay

TNF-α was measured by a sandwich-type ELISA using two monoclonal antibodies (Department of Immune Reagents, Central

© Cancer Research Campaign 1997

Laboratory of Blood Transfusion, Amsterdam, Netherlands) raised against rhTNF-α (courtesy of Dr A Creasey, Chiron, Emeryville, CA, USA). One MAb (MAb CLB-TNFα-7) was used for coating at a concentration of 2 μg $ml^{-1}$; the second MAb (MAb CLB-TNFα-5) was biotinylated and used in combination with streptavidin poly-horseradish peroxidase conjugate to detect bound TNF-α. Stimulated human mononuclear cell supernatant was used as a standard for comparison with purified rhTNF-α. Results were expressed as pg $ml^{-1}$ by reference to this standard.

### Histology

Multiple liver biopsies were taken before and directly after IHP and upon sacrifice at 4–6 weeks post-operatively. The tissue samples were fixed in formaldehyde and embedded in paraffin. Five-micrometre sections were stained with haematoxylin and eosin (HE). In addition, samples were taken from all animals in preparation for electronmicroscopy (EM).

### Statistics

Comparisons within and between groups were made by analysis of variance for repeated measurements (ANOVA) or by the *t*-test where appropriate. Correlations between maximum or minimum levels of parameters were calculated as Spearmann's rank correlations. The significance level was taken as a probability (two-sided) of $< 0.05$.

### RESULTS

The duration of the operation ranged from 4 to 7 h (median 6 h). In all animals, a stable perfusion was achieved with no apparent leakage as demonstrated by the fluorescein dye injection. Further technical details are summarized in Table 1. As indicated by the oxygen saturation levels in the perfusate, adequate tissue perfusion was attained in all cases. In addition, the measured flow rates in the PV did not differ significantly before and after IHP in all groups. Median blood loss was 500 ml (range 300–1500 ml), including blood lost in the perfusion circuit. All pigs survived the operation. One animal in the TNF-alone group died on the first post-operative day. At necropsy, clear, serosanguinous fluid was observed in the abdomen without evidence of portal hypertension/thrombosis or surgical haemorrhage. One pig in the TNF/melphalan group underwent relaparotomy for hernia cicatricalis 2 weeks after IHP; one pig in the TNF-alone group developed pneumonia with elevated leucocyte counts 4 weeks after perfusion. At the time of necropsy, all remaining animals were in good general condition, with weights ranging from 30 to 40 kg. In fact, 4 weeks after IHP, all surviving animals had gained weight. Weight gains did not differ significantly between groups. Macroscopic post-mortem examination did not reveal any intra-abdominal or intrathoracic abnormalities.

In all animals, IHP resulted in significant elevations of ASAT, ALAT, LDH and alkaline phosphatase levels, with peak values occurring on day 1 post-operatively (Figure 1). Transaminase levels returned to normal within the first 7–10 post-operative days, while alkaline phosphatase and LDH remained slightly elevated throughout the observation period. Total bilirubin values remained within the normal range (Figure 1), as did the serum values of urea, γ-GT and creatinin (data not shown). There were no significant differences in peak values or kinetics between the three groups. In all groups, serum albumin levels decreased to a nadir of approximately 22 g $l^{-1}$ on the first post-operative day and returned to normal values within the next 7–14 days, (Figure 1). Haemoglobin and haematocrit remained normal throughout the follow-up period (data not shown). In contrast, platelet counts decreased slightly, but not significantly, during the first post-operative day and normalized within 3–7 days.

**Figure 2** Perfusate (**A**) and systemic (**B**) TNF-α levels (pg $ml^{-1}$) as a function of time before, during and after IHP in pigs. -■-, TNF; *, TNF/melphalan; -■-, control

TNF-α levels in the perfusate of the pigs in the TNF-alone group increased to a median of $5.0 \times 10^6$ pg $ml^{-1}$ (range 4.9–$6.3 \times 10^6$); compared with $5.2 \times 10^6$ pg $ml^{-1}$ (5.1–6.6 pg $ml^{-1}$) in the TNF/melphalan group. These perfusate TNF-α levels remained virtually stable throughout the 1-h perfusion period. Perfusate TNF-α levels in the control group remained normal (i.e. $< 5$ pg $ml^{-1}$) during IHP (Figure 2). At $t = 0$ (i.e. at the beginning of the perfusion), all animals displayed normal systemic TNF-α levels. During IHP, systemic TNF-α levels in the control group increased to a median of 12 pg $ml^{-1}$ (8.9–15 pg $ml^{-1}$) at $t = 60$ min, compared with 76 pg $ml^{-1}$ (41–120 pg $ml^{-1}$) in the TNF-alone group and 139 pg $ml^{-1}$ (34–197 pg $ml^{-1}$) in the TNF/melphalan group. These figures indicate that, in both experimental groups, cumulative systemic leakage of TNF-α from the perfusate was less than 0.02% during the 60-min perfusion. However, following washout and decannulation at the end of the perfusion, systemic TNF-α levels increased significantly in the TNF-alone group

*© Cancer Research Campaign 1997*

and the TNF/melphalan group, with median peak levels of $3.2 \times 10^3$ pg $ml^{-1}$ and $17 \times 10^3$ pg $ml^{-1}$ respectively (Figure 2). These peak levels occurred between 1 and 30 min (median 10 min) after washout and returned to normal within 480 min after IHP. Again, there were no significant differences between the two experimental groups. Systemic post-perfusion TNF-α levels in the control animals rose slightly, but not significantly, to a maximum value of 26 pg $ml^{-1}$ at $t = 60$ min after washout. None of the biliary samples evaluated contained detectable levels of TNF-α.

Compared with pre-perfusion histology, microscopic examination of HE-stained sections taken directly after perfusion showed mild sinusoidal dilatation as well as septal oedema with sporadic intraseptal polymorphonuclear cell (PMN) infiltration. These findings were documented in all animals, including the controls. There was no apparent hepatocellular damage or parenchymal necrosis. At 4–6 weeks after IHP, all microscopical sections revealed normal pig liver histology (on both HE and EM), with the exception of sporadic PMN infiltrates in the liver parenchyma. The septal oedema and sporadic septal infiltration had disappeared in all specimens investigated. Again, these findings were similar in all three groups.

### DISCUSSION

The data presented here demonstrate that, in the pig model used, hyperthermic isolated perfusion of the liver via both the HA and the PV is technically feasible and appears to be a safe procedure. Nevertheless, the current IHP technique still involves a large operation, as illustrated by the median duration of 6 h and the one post-operative death. Additional modifications, including the use of balloon catheters, are therefore being studied at present. Temporary exposure of normal porcine liver parenchyma to high-dose rhTNF-α, with and without melphalan, in combination with hyperthermia is well tolerated and results in mild, transient hepatotoxicity. This was illustrated by early elevation of liver enzyme levels, followed by a spontaneous return to normal levels. On histological examination, immediate post-perfusion changes included sinusoidal dilatation and mild septal oedema, without any signs of hepatocellular injury. Sections taken 4–6 weeks after IHP revealed sporadic, periportal infiltrates in otherwise normal hepatic parenchyma. Most biochemical and histological alterations following IHP were similar in both control and experimental animals. This suggests that the mild hepatotoxic phenomena observed were primarily caused by the IHP procedure itself and that the addition of the drugs used, in particular rhTNF-α, does not lead to additional hepatotoxicity. These findings are in agreement with those reported on IHP with hyperthermia and/or standard chemotherapeutics (Skibba, 1983, 1988; Sindelar, 1985; Van de Velde, 1986; Aigner, 1988; Hafstrom 1994).

Complete vascular isolation of the liver during IHP is essential to avoid systemic exposure to high doses of antitumoral agents. Previous studies on IHP in large animals, using somewhat different methodologies, have mentioned technical difficulties resulting in incomplete vascular isolation and systemic leakage of drugs. Van de Velde et al (1986) reported leakage in 3 out of 15 pigs treated with IHP, whereas Sindelar et al (1985) encountered incomplete vascular isolation in 2 out of 10 pigs, resulting in severe systemic drug-associated toxicity and death. In these studies, either a passive external or an internal venous shunt was employed to drain distal portal and lower body blood. In view of their findings, we modified the IHP technique in an attempt to minimize leakage. This modification involved the introduction of a separate, second active circuit which consisted of a pump-aided, extracorporeal veno-venous bypass shunt (VVB) connecting cannulas in the distal PV and infrarenal IVC with the external jugular vein. Besides simplifying the hepatic perfusion circuit in this manner (as opposed to internal venous shunts), the VVB has the additional advantage of more efficiently shunting blood from the lower body, kidneys and intestines to the heart. As a result, the cardiac venous return increases, thereby augmenting haemodynamic stability throughout the procedure. In fact, we did not observe any haemodynamic instability during our experiments in pigs, generally considered to be haemodynamically sensitive. Moreover, we have been able to detect that there was no significant leakage from the liver perfusion circuit to the systemic circulation. This was achieved using either of two qualitative methods, i.e. observing fluorescent dye distribution or monitoring perfusate reservoir levels. This was confirmed in a quantitative manner by analysing, during the vascular isolation period, systemic levels of TNF-α, which remained approximately four orders of magnitude lower than perfusate levels. In addition, all pigs survived the procedure and no animal demonstrated any of the known systemic side-effects of rhTNF-α in pigs during and after IHP (Leighton, 1991; Truog, 1992).

However, following IHP and washout, an additional rise in systemic TNF-α levels was seen upon restoration of vascular continuity. Although well below toxic concentrations of rhTNF in the pig, this phenomenon still has to be accounted for. It is possible that the washout procedure was not sufficiently effective in removing all remaining TNF-α from the perfusate. This may be particularly true in the non-tumour-bearing pig liver, in which virtually no TNF-uptake was observed during IHP, as judged by perfusate TNF-α levels (Figure 2).

There is no consensus on the route of infusion (HA *vs* PV *vs* both). Normal hepatic parenchyma receives most of its blood supply from branches of the PV and to a much lesser extent from the HA. In contrast, the blood supply of hepatic metastases is reported to rely almost entirely on the HA (Strohmeyer, 1986). Consequently, most regional approaches have been made using the HA. More recently, however, attention has been drawn to the PV as very small liver tumours (< 5 mm), as well as the outer rim of larger hepatic metastases, are fed mainly by portal branches (Archer, 1989). In addition, most colorectal tumours are drained via the PV, suggesting that spreading tumour cells will first proliferate in the portal system. Thus, by using the HA as well as the PV, drugs will reach both established and newly formed (micro) metastases. Taking this into consideration, we performed IHP via both the HA as well as the PV. However, as most normal hepatic parenchyme tissue is supplied primarily by the PV, it could be speculated that infusion via the PV might induce significant hepatotoxicity. Indeed, Boddie et al (1979) performed IHP solely via the PV and demonstrated significant hepatic damage. In accordance with most other reports on IHP, we have not been able to confirm these findings (Skibba, 1983, 1988; Sindelar, 1985; Van de Velde, 1986; Aigner, 1988; Hafstrom, 1994).

At present, it is unknown which drug, or combination of drugs, would provide antitumoral efficacy in the IHP setting. TNF-α with and without melphalan was selected for this study based on its clinical success (100% limb salvage and a 90% overall response) in isolated limb perfusions for irresectable melanoma and sarcoma (Eggermont, 1994; Liénard, 1994). As at least part of the anti-tumour effect of TNF-α relies on the destruction of the tumour-associated vessels, irrespective of tumour histology (Watanabe,

© Cancer Research Campaign 1997

1988; Renard, 1994; Cid, 1994), we reasoned that this combination might well be effective against colorectal hepatic metastases. Indeed, Van der Schelling et al (1992) have recently shown that intratumoral administration of rhTNF-α, under ultrasonographic guidance, was able to stabilize disease in eight patients with hepatic metastases at the cost of minimal systemic symptoms. As demonstrated by Mavligit et al (1992), HA infusion of rhTNF-α permits a more than sixfold dose increase of the maximum tolerated systemic (i.v.) dose before adverse systemic side-effects are noted. In this setting, TNF-α was found to induce tumour regression in approximately 30% of patients with irresectable colorectal liver metastases (Mavligt, 1992). Because of the synergy between melphalan and TNF-α, as demonstrated in earlier reports (Eggermont, 1996*a*,*b*; Liénard, 1994), melphalan was chosen over 5-FU, the drug most frequently used in conventional regimens against colorectal (liver) metastases. For reasons of comprehensiveness, we also performed IHP with TNF-α and 5-FU in two pigs. No mortality was encountered, and (hepatic) response patterns were identical to the ones described above (data not shown).

On the other hand, Kahky et al (1990) have shown that intraportal administration of 100 μg $kg^{-1}$ $day^{-1}$ rhTNF-α results in 100% mortality in rats. Histological examination in their study revealed mild passive congestion of the liver combined with severe pulmonary oedema. As systemic administration of the same dose of rhTNF-α did not result in any deaths, it is unlikely that the high mortality after intraportal injection was caused solely by TNF-α (Kahky, 1990). TNF-α has been documented to induce the production of various cytokines (including IL-1, IL-6 and TNF-α) by macrophages (i.e. Kupffer cells) (Shirahama, 1988; Busam, 1990). As the vast majority of hepatic Kupffer cells are situated in the (peri) portal area, such a secondary cytokine release might explain the observed mortality. As far as we can judge, IHP with rhTNF-α and melphalan in the healthy pig does not lead to such dramatic cytokine-related side-effects.

In conclusion, hyperthermic IHP with rhTNF-α and melphalan in pigs is technically feasible, resulting in minimal systemic leakage of drugs and mild, transient hepatotoxicity. The addition of rhTNF-α and melphalan in the perfusate does not lead to additional hepatotoxic side-effects. As pig liver physiology is similar to humans, IHP with rhTNF-α and melphalan should be considered for phase I evaluation in patients with irresectable hepatic malignancy.

### ACKNOWLEDGEMENTS

The authors gratefully acknowledge Janny de Kam, Enno Collij, Henk Dronk and Rob Meijer for their excellent technical assistance during the operative procedures. They are also indebted to the Department of Extracorporeal Circulation of the University Hospital Rotterdam (Head: Mrs M Wijers) for their superb perfusionists' skills and cooperation.

### REFERENCES

Aigner KR, Walther H, Tonn JC, Wenzl A, Merker G and Schwemmle K (1982) Die isolierte Leberperfusion mit 5-Fluorouracil (5-FU) beim Menschen. *Chirurg* **53**: 571–573

Aigner KR (1988) Isolated liver perfusion: 5-year results. *Reg Cancer Treat* **1**: 11–20

Alexander RB and Rosenberg SA (1991) Tumour necrosis factor: clinical application. In *Biologic Therapy of Cancer*, De Vita Jr VT, Hellman S and Rosenberg SA. (eds), pp. 378–392. JB Lippincott: Philadelphia.

Archer SG and Gray BN (1989) Vascularization of small liver metastases. *Br J Surg* **76**: 545–548

Asher A, Mule JJ, Reichert CM, Shipant E and Rosenberg SA (1987) Studies on the anti-tumor efficacy of systemically administered recombinant tumor necrosis factor against several murine tumors in vivo. *J Immunol* **138**: 963–974

Bengmark S and Hafstrom L (1969) The natural history of primary and secondary tumours of the liver. I. The prognosis for patients with hepatic metastases from colonic and renal carcinoma by laparotomy. *Cancer* **23**: 198–202

Blick M, Sherwin SA, Rosenblum M and Gutterman J (1987) Phase I study of recombinant tumor necrosis factor in cancer patients. *Cancer Res* **47**: 2986–2989

Boddie AW Jr, Booker L, Mullins JD, Buckley CJ and McBride CM (1979) Hepatic hyperthermia by total isolation and regional perfusion in vivo. *J Surg Res* **26**: 447–457

Busam KJ, Baver TM, Baver J, Gerok W and Decker K (1990) Interleukin-6 release by rat liver macrophages. *J Hepatol* **11**: 367–373

Cady B and Stone M (1991) The role of surgical resection of liver metastases in colorectal carcinoma. *Semin Oncol* **18**: 4399–406

Canellos GP (1987) The case for high-dose chemotherapy: is it chemotherapy's last gamble? *Eur J Cancer Clin Oncol* **23**: 351–355

Chang AE, Schneider PD, Sugarbaker PH, Simpson C, Culnane M and Steinberg SM (1987) A prospective randomized trial of regional versus systemic continuous 5-fluorodeoxyuridine chemotherapy in the treatment of colorectal liver metastases. *Ann Surg* **206**: 685–693

Cid MC, Kleinman HK, Grant DS, Schnaper HW, Favci AS and Hoffman GS (1994) Estradiol enhances leucocyte binding to tumor necrosis factor (TNF)-stimulated endothelial cells via an increase in TNF-induced adhesion molecules E-selectin, intercellular adhesion molecule type 1, and vascular cell adhesion molecule type 1. *J Clin Invest* **93**: 17–25

De Brauw LM, Van De Velde CJH, Tjaden UR, De Bryijn EA, Bell AURJ, Hermans J and Zwaveling A (1988) In vivo isolated liver perfusion technique in a rat hepatic metastasis model: 5-fluorouracil concentrations in tumor tissue. *J Surg Res* **44**: 137–145

De Takats PG, Kerr DJ, Poole CJ, Warren HW and McArdle CS (1994) Hepatic arterial chemotherapy for metastatic colorectal carcinoma (review). *Br J Cancer* **69**: 372–378

Eggermont AMM, Schraffordt Koops H, Lienard D, Kroon BBR, Van Geel AN, Hoekstra HJ and Lejeune FJ (1994) High-dose Tumor Necrosis Factor-α in combination with Interferon-γ and Melphalan in isolated perfusion of the limb for irresectable soft tissue sarcomas: a highly effective approach to achieve limb salvage. In *Cytokines in Cancer Therapy*, Bergmann L and Mitrou PS. (eds), pp. 81–88. Contrib Oncol Vol. 46. Karger: Basle

Eggermont AMM, Schraffordt Koops H, Lienard D, Kroon BBR, Van Geel AN, Hoekstra HJ and Lejeune FJ (1996*a*). Isolated limb perfusion with high dose tumor necrosis factor-α in combination with IFNγ- and melphalan for irresectable extremity soft tissue sarcomas: a multicenter trial. *J Clin Oncol* **14**: 2653–2665

Eggermont AMM, Schraffordt Koops H, Klausner J, Kroon BBR, Schlag PM, Lienard D, Van Geel AN, Hoekstra HJ, Meller I, Nieweg OE, Kettelhack C, Ben-Ari G, Pector JC and Lejeune FJ (1996*b*) Isolated limb perfusion with tumor necrosis factor-a and melphalan in 186 patients with locally advanced extremity sarcomas: the cumulative multicentre European experience. *Ann Surgery* **224**: 756–765

Feinberg B, Kurzrock R, Talpa ZM, Blick M, Saks S and Gutterman JU (1988) A phase I trial of intravenously administered recombinant tumor necrosis factor in patients with advanced cancer. *J Clin Oncol* **6**: 1328–1334

Frei E and Canellos GP (1980) Dose: a critical factor in cancer chemotherapy. *Am J Med* **69**: 585–594

Genari L (1992) Liver metastases: a many-sided therapeutical problem. *Hepatogastroenterology* **39**: 5–9

Greenway B (1988) Hepatic metastases from colorectal cancer: resection or not. *Br J Surg* **75**: 513–551

Hafstrom LR, Holmberg SB, Nazedi PL, Bengtsson A, Tidebrandt G and Schertsen TS (1994) Isolated hyperthermic liver perfusion with chemotherapy for liver malignancy. *Surg Oncol* **3**: 103–108

Hughes KS, Simon R, Songhorabodj S, Adson MA, Ilstrup DM, Fortner JG, Maclean BJ, Foster JH, Daly JM, Fitzherbert D and Sugarbalrer PH (1986) Resection of the liver for colorectal carcinoma metastases: a multiinstitutional study of patterns of recurrence. *Surgery* **100**: 278

Jaattela M (1991) Biologic activities and mechanisms of action of tumor necrosis factor α/ cachectin. *Lab Invest* **64**: 724–742

Kahky MP, Daniel CO, Cruz AB and Gaskill III HV (1990) Portal infusion of tumor necrosis factor increases mortality in rats. *J Surg Res* **49**: 138–145

 © Cancer Research Campaign 1997

Kemeny N (1983) The systemic chemotherapy of hepatic metatases. *Semin Oncol* **10**: 148–155

Kemeny N, Daly J, Reichman B, Geller N, Botet J and Oderman P (1987) Intrahepatic or systemic infusion of fluorodeoxyuridine in patients with liver metastases from colorectal carcinoma. *Ann Int Med* **107**: 459–465

Leighton TA Averbook AW, Klein SR and Bongard FS (1991) Time-course of cardiopulmonary effects of tumor necrosis factor and endotoxin are similar. *Am Surg* **57**: 836–842

Lienard D, Eggermont AMM, Schraffordt Koops H, Kroon BBR, Rosen Kaimer F, Autier P and Lejeune FJ (1994) Isolated perfusion of the limb with high-dose tumour necrosis factor-alpha (TNF-α), interferon-gamma (IFN-γ) and melphalan for melanoma stage III. Results of a multi-center pilot study. *Melanoma Res* **4**: 21–26

Marinelli A, Van Dierendonck JH, Van Brakel GM, Irth H, Kuppen PJK, Tjaden VR and Van De Velde CJH (1991) Increasing the effective concentration of melphalan in experimental rat liver tumours: comparison of isolated liver perfusion and hepatic artery infusion. *Br J Cancer* **64**: 1069–1075

Mavligit GM, Zukiwskj AA, Charnsangavej C, Carrasco CH, Wallace S and Gutterman GM (1992) Hepatic arterial infusion with human tumor necrosis factor in patients with liver metastases. *Cancer* **69**: 557–561

Pentecost MJ (1993) Transcatheter treatment of hepatic metastases. *Am J Roentgenol* **160**: 1171–1175

Que FG and Nagorney DM (1994) Resection of 'recurrent' colorectal metastases to the liver. *Br J Surg* **81**: 255–258

Radnell M, Jeppsson B and Bengmark S (1990) A technique for isolated liver perfusion in the rat with survival and results of cytotoxic drug perfusion on liver tumor growth. *J Surg Res* **49**: 394–399

Renard N, Lienard D, Lespagnard L, Eggermont A, Heimann R and Lejeune F (1994) Early endothelium activation and polymorphonuclear cell invasion precede specific necrosis of human melanoma and sarcoma treated by intravascular high-dose tumour necrosis factor alpha (rTNFα). *Int J Cancer* **57**: 656–663

Scheele J, Strangl R and Altendorf-Hofmann A (1990) Hepatic metastases from colorectal carcinoma: impact of surgical resection on the natural history. *Br J Surg* **77**: 1241–1246

Shirahama M, Ishibasi H, Tsuchiya Y, Kurokawa S, Okomura Y and Niho Y (1988) Kinetics and parameters of the induction of interleukin-1 secretion by rat Kupffer cells. *J Clin Lab Immunol* **27**: 127–132

Sidhu RS and Bollon AP (1993) Tumor necrosis factor activities and cancer therapy – a perspective. *Pharmac Ther* **57**: 79–128

Sindelar WF (1985) Isolation-perfusion of the liver with 5-fluorouracil. *Ann Surg* **201**: 337–343

Skibba JL and Condon RE (1983) Hyperthermic isolation perfusion in vivo of the canine liver. *Cancer* **51**: 1303–1309

Skibba JL, Quebbeman EJ, Komorowski RA and Thorsen KM (1988) Clinical results of hyperthermic liver perfusion for cancer in the liver. *Contr Oncol* **29**: 222–228

Starzl TA (1990) Liver transplantation: a 30-year perspective. Part I. *Curr Probl Surg* **27**: 73–76

Strangl R, Altendorf-Hofman A, Charnley RM and Scheele J (1994) Factors influencing the natural history of colorectal liver metastases. *Lancet* **343**: 1405–1410

Strohmeyer T, Haugeberg G and Lierse W (1986) Vaskularisation von Lebermetastasen: eine korrosionsanatomische Studie. *Acta Anat* **126**: 172–176

Sugihara K, Hojo K, Moriya Y, Yamasaki S, Kosuget and Takayama T (1993) Pattern of recurrence after hepatic resection for colorectal metastases. *Br J Surg* **80**: 1032–1035

Sullivan RD, Norcoss JW and Watkins E (1964) Chemotherapy of metastatic liver cancer by prolonged hepatic artery infusion. *N Engl J Med* **270**: 321–327

Truog WE, Gibson RL, Henderson WR, Redding GJ and Standaert TA (1992) Effect of pentoxifylline on cytokine- and eicosanoid-induced acute pulmonary hypertension in piglets. *Pediatr Res* **31**: 163–169

Van De Velde CJH, Kothuis BJL, Barenbrug HWM, Jongejan N, Runia RD, De Brauw LM and Zwaveling A (1986) A successful technique of in vivo isolated liver perfusion in pigs. *J Surg Res* **41**: 593–599

Van Der Schelling GP, Ijzermans JNM, Kok TC, Schering M, Marquet RL, Splinter TAW and Jeekel J (1992) A phase I study of local treatment of liver metastases with recombinant tumor necrosis factor. *Eur J Cancer* **28A**: 1073–1078

Van Ooijen B, Wiggers T, Meyer S, Van Der Heijde MN, Sloof MJH, Van De Velde CJH, Obertop H, Gouma DJ, Bruggink EDM, Lang JF, Munting DK, Rutten APM, Rutten H, de Vries JE, Groot G, Zoutmulder FAN and van Putten WLJ (1992) Hepatic resections for colorectal metastases in The Netherlands. A multi-institutional 10-year study. *Cancer* **70**: 28–34

Wagner JS, Adson MA, Van Heerden JA, Adson MH and Ilstrup DM (1984) The natural history of hepatic metastases from colorectal origin. A comparison with resective treatment. *Ann Surg* **199**: 502–508

Watanabe N, Niitsu Y, Umeno H, Kuriyama H, Neda H, Yamauchi N, Maeda M and Urushizaki I (1988) Toxic effect of TNF on tumor vasculature in mice. *Cancer Res* **49**: 2179–2183

© Cancer Research Campaign 1997